# Ethyl­enediaminium dinicotinate

**DOI:** 10.1107/S1600536811023877

**Published:** 2011-06-25

**Authors:** Liang Zhao, Li-ping Feng

**Affiliations:** a Department of Chemical & Environmental Engineering, Anyang Institute of Technology, Anyang 455000, People’s Republic of China

## Abstract

In the title compound, C_2_H_10_N_2_^2+^·2C_6_H_4_NO_2_^−^, the cation lies on an inversion centre. The asymmetric unit is composed of one nicotinate anion and one half ethyl­enediaminium cation. All the amino H atoms are involved in N—H⋯O and N—H⋯N hydrogen bonds. These hydrogen bonds link the ionic units into a three-dimensional network. In addition, π–π inter­actions between pyridine rings [centroid–centroid distance = 3.6037 (7) Å] further stabilize the crystal structure.

## Related literature

For applications of amino compounds, see: Fu *et al.* (2010 ▶); Aminabhavi *et al.* (1986 ▶).
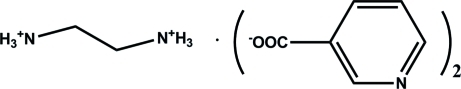


## Experimental

### 

#### Crystal data

C_2_H_10_N_2_^2+^·2C_6_H_4_NO_2_^−^
*M* *_r_* = 306.32
Monoclinic, 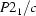

*a* = 6.2953 (13) Å
*b* = 16.835 (3) Å
*c* = 6.8288 (14) Å
β = 102.03 (3)°
*V* = 707.8 (3) Å^3^
*Z* = 2
Mo *K*α radiation
μ = 0.11 mm^−1^
*T* = 298 K
0.30 × 0.05 × 0.05 mm

#### Data collection

Rigaku Mercury2 diffractometer
Absorption correction: multi-scan (*CrystalClear*; Rigaku, 2005 ▶) *T*_min_ = 0.910, *T*_max_ = 1.000
7181 measured reflections
1618 independent reflections
1162 reflections with *I* > 2σ(*I*)
*R*_int_ = 0.056

#### Refinement

*R*[*F* ^2^ > 2σ(*F* ^2^)] = 0.062
*wR*(*F* ^2^) = 0.147
*S* = 1.08
1618 reflections
101 parameters
H-atom parameters constrained
Δρ_max_ = 0.23 e Å^−3^
Δρ_min_ = −0.25 e Å^−3^

### 

Data collection: *CrystalClear* (Rigaku, 2005 ▶); cell refinement: *CrystalClear*; data reduction: *CrystalClear*; program(s) used to solve structure: *SHELXS97* (Sheldrick, 2008 ▶); program(s) used to refine structure: *SHELXL97* (Sheldrick, 2008 ▶); molecular graphics: *SHELXTL* (Sheldrick, 2008 ▶); software used to prepare material for publication: *SHELXTL*.

## Supplementary Material

Crystal structure: contains datablock(s) I, global. DOI: 10.1107/S1600536811023877/bx2355sup1.cif

Structure factors: contains datablock(s) I. DOI: 10.1107/S1600536811023877/bx2355Isup2.hkl

Supplementary material file. DOI: 10.1107/S1600536811023877/bx2355Isup3.cml

Additional supplementary materials:  crystallographic information; 3D view; checkCIF report

## Figures and Tables

**Table 1:** Hydrogen-bond geometry (Å, °)

*D*—H⋯*A*	*D*—H	H⋯*A*	*D*⋯*A*	*D*—H⋯*A*
N1—H1*B*⋯N2^i^	0.90	2.24	2.971 (3)	138
N1—H1*C*⋯O1^ii^	0.90	1.85	2.729 (3)	164
N1—H1*A*⋯O2	0.90	1.83	2.711 (3)	166

Symmetry codes: (i) 
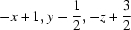
; (ii) 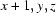.

**Fig. 1.**
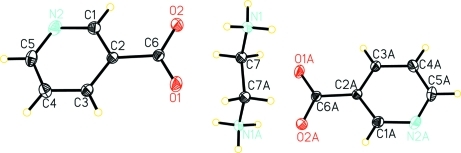
Molecular view of the title compound with the atomic numbering scheme. Displacement ellipsoids are drawn at the 30% probability level.

**Fig. 2.**
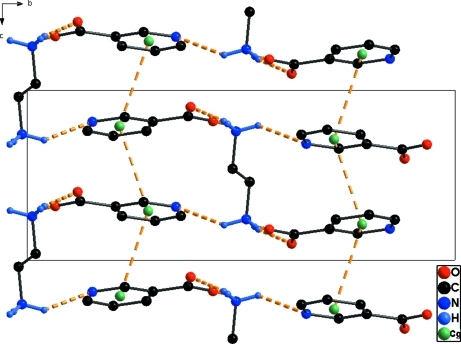
The crystal packing of the title compound viewed along the c axis showing the one-dimensionnal hydrogen bondings chain (dashed line). Hydrogen atoms not involved in hydrogen bonding have been omitted for clarity.

## supplementary crystallographic information

### Comment

The amino derivatives have found wide range of applications in material science, such as magnetic, fluorescent and dielectric behaviors, and there has been an increased interest motif in the preparation of amino cocrystal compounds (Aminabhavi *et al.,* 1986; Fu, *et al.* 2010). We report here the crystal structure of the title compound. In the title compound, [(C_2_H_10_N_2_)(C_6_H_4_NO_2_)_2_], the cation lies on inversion centre. The asymmetric unit is composed of one nicotinate anion and one-half ethylenediaminium cation, (Fig.1). Both the amine N atoms of the ethylenediaminium cation are protonated. The geometric parameters are in the normal range. In the crystal structure, all the amino group H atoms are involved in N—H···O and N—H···N hydrogen bonds (Table1). These hydrogen bonds link the ionic units into a three-dimentional network. In addition, the pyridine rings π–π (centroid-to-centroid distance = 3.6037 (7) Å, symmetry code: x, 1/2-y, 1/2+z) interactions further stabilized the structure (Fig. 2).

### Experimental

A mixture of ethylenediamine (0.4 mmol) and nicotinic acid (0.8 mmol) were dissolved in distilled water (10 ml). Colorless block crystals suitable for X-ray analysis were obtained after 3 days.

### Refinement

All H atoms attached to C atoms were fixed geometrically and treated as riding with C— H = 0.97 Å(methylene) and C— H = 0.93 Å(aromatic) with *U*_iso_(H) = 1.2*U*_eq_(C). The positional parameters of the H atoms (N1) were refined freely, in the last stage of the refinement, it were restrained with the H—N = 0.90 (2) Å, with *U_iso_*(H) = 1.5*U_eq_*(N).

### Crystal data

**Table:**

C_2_H_10_N_2_^2+^·2C_6_H_4_NO_2_^−^	*F*(000) = 324
*M**_r_* = 306.32	*D*_x_ = 1.437 Mg m^−^^3^
Monoclinic, *P*2_1_/*c*	Mo *K*α radiation, λ = 0.71073 Å
Hall symbol: -P 2ybc	Cell parameters from 1618 reflections
*a* = 6.2953 (13) Å	θ = 3.3–27.5°
*b* = 16.835 (3) Å	µ = 0.11 mm^−^^1^
*c* = 6.8288 (14) Å	*T* = 298 K
β = 102.03 (3)°	Block, colourless
*V* = 707.8 (3) Å^3^	0.30 × 0.05 × 0.05 mm
*Z* = 2	

### Data collection

**Table:**

Rigaku Mercury2 diffractometer	1618 independent reflections
Radiation source: fine-focus sealed tube	1162 reflections with *I* > 2σ(*I*)
graphite	*R*_int_ = 0.056
Detector resolution: 13.6612 pixels mm^-1^	θ_max_ = 27.5°, θ_min_ = 3.3°
CCD profile fitting scans	*h* = −7→8
Absorption correction: multi-scan (*CrystalClear*; Rigaku, 2005)	*k* = −21→21
*T*_min_ = 0.910, *T*_max_ = 1.000	*l* = −8→8
7181 measured reflections	

### Refinement

**Table:**

Refinement on *F*^2^	Secondary atom site location: difference Fourier map
Least-squares matrix: full	Hydrogen site location: inferred from neighbouring sites
*R*[*F*^2^ > 2σ(*F*^2^)] = 0.062	H-atom parameters constrained
*wR*(*F*^2^) = 0.147	*w* = 1/[σ^2^(*F*_o_^2^) + (0.0557*P*)^2^ + 0.4625*P*] where *P* = (*F*_o_^2^ + 2*F*_c_^2^)/3
*S* = 1.08	(Δ/σ)_max_ < 0.001
1618 reflections	Δρ_max_ = 0.23 e Å^−^^3^
101 parameters	Δρ_min_ = −0.25 e Å^−^^3^
0 restraints	Extinction correction: *SHELXL97* (Sheldrick, 2008), Fc^*^=kFc[1+0.001xFc^2^λ^3^/sin(2θ)]^-1/4^
Primary atom site location: structure-invariant direct methods	Extinction coefficient: 0.032 (6)

### Special details

**Table:**

Geometry. All esds (except the esd in the dihedral angle between two l.s. planes) are estimated using the full covariance matrix. The cell esds are taken into account individually in the estimation of esds in distances, angles and torsion angles; correlations between esds in cell parameters are only used when they are defined by crystal symmetry. An approximate (isotropic) treatment of cell esds is used for estimating esds involving l.s. planes.
Refinement. Refinement of F^2^ against ALL reflections. The weighted R-factor wR and goodness of fit S are based on F^2^, conventional R-factors R are based on F, with F set to zero for negative F^2^. The threshold expression of F^2^ > 2sigma(F^2^) is used only for calculating R-factors(gt) etc. and is not relevant to the choice of reflections for refinement. R-factors based on F^2^ are statistically about twice as large as those based on F, and R- factors based on ALL data will be even larger.

### Fractional atomic coordinates and isotropic or equivalent isotropic displacement parameters (Å^2^)

**Table:**

	*x*	*y*	*z*	*U*_iso_*/*U*_eq_	
O1	0.0882 (3)	0.56578 (10)	0.8189 (3)	0.0407 (5)	
O2	0.4172 (3)	0.61799 (10)	0.9019 (3)	0.0415 (5)	
C2	0.1214 (3)	0.70652 (13)	0.8166 (3)	0.0226 (5)	
N1	0.6707 (3)	0.51198 (11)	0.7583 (3)	0.0284 (5)	
H1A	0.5813	0.5405	0.8182	0.043*	
H1B	0.6812	0.4603	0.7926	0.043*	
H1C	0.8057	0.5313	0.8016	0.043*	
N2	0.1923 (3)	0.84765 (12)	0.8146 (3)	0.0366 (5)	
C3	−0.0997 (4)	0.72136 (13)	0.7603 (4)	0.0284 (5)	
H3A	−0.1982	0.6795	0.7416	0.034*	
C5	−0.0222 (4)	0.85903 (14)	0.7611 (4)	0.0346 (6)	
H5A	−0.0727	0.9110	0.7423	0.042*	
C1	0.2585 (4)	0.77203 (14)	0.8423 (4)	0.0308 (6)	
H1D	0.4069	0.7628	0.8816	0.037*	
C6	0.2143 (3)	0.62372 (13)	0.8475 (3)	0.0248 (5)	
C7	0.6080 (4)	0.52001 (14)	0.5385 (3)	0.0278 (5)	
H7B	0.7182	0.4959	0.4773	0.033*	
H7A	0.5977	0.5758	0.5025	0.033*	
C4	−0.1726 (4)	0.79878 (14)	0.7320 (4)	0.0326 (6)	
H4A	−0.3202	0.8098	0.6942	0.039*	

### Atomic displacement parameters (Å^2^)

**Table:**

	*U*^11^	*U*^22^	*U*^33^	*U*^12^	*U*^13^	*U*^23^
O1	0.0329 (10)	0.0209 (9)	0.0674 (13)	−0.0021 (7)	0.0086 (9)	−0.0007 (8)
O2	0.0237 (9)	0.0340 (10)	0.0633 (13)	0.0046 (7)	0.0006 (8)	−0.0115 (9)
C2	0.0247 (11)	0.0225 (11)	0.0210 (11)	−0.0010 (9)	0.0058 (9)	−0.0012 (9)
N1	0.0268 (10)	0.0222 (10)	0.0342 (11)	0.0025 (8)	0.0016 (8)	−0.0010 (8)
N2	0.0402 (12)	0.0247 (11)	0.0466 (13)	−0.0054 (9)	0.0128 (10)	−0.0029 (9)
C3	0.0278 (12)	0.0223 (12)	0.0346 (13)	−0.0024 (9)	0.0050 (10)	−0.0004 (10)
C5	0.0485 (15)	0.0214 (13)	0.0370 (14)	0.0037 (11)	0.0158 (12)	0.0031 (10)
C1	0.0262 (12)	0.0286 (13)	0.0383 (14)	−0.0031 (10)	0.0081 (10)	−0.0022 (10)
C6	0.0257 (12)	0.0234 (12)	0.0253 (12)	0.0000 (9)	0.0057 (9)	−0.0028 (9)
C7	0.0266 (12)	0.0236 (12)	0.0326 (13)	−0.0029 (9)	0.0048 (10)	0.0022 (10)
C4	0.0299 (13)	0.0308 (13)	0.0370 (14)	0.0047 (10)	0.0069 (11)	0.0044 (11)

### Geometric parameters (Å, °)

**Table:**

O1—C6	1.247 (3)	N2—C1	1.341 (3)
O2—C6	1.257 (3)	C3—C4	1.382 (3)
C2—C3	1.387 (3)	C3—H3A	0.9300
C2—C1	1.389 (3)	C5—C4	1.373 (3)
C2—C6	1.509 (3)	C5—H5A	0.9300
N1—C7	1.476 (3)	C1—H1D	0.9300
N1—H1A	0.9005	C7—C7^i^	1.510 (4)
N1—H1B	0.9004	C7—H7B	0.9700
N1—H1C	0.9004	C7—H7A	0.9700
N2—C5	1.337 (3)	C4—H4A	0.9300
			
C3—C2—C1	116.9 (2)	N2—C1—C2	124.7 (2)
C3—C2—C6	122.82 (19)	N2—C1—H1D	117.6
C1—C2—C6	120.2 (2)	C2—C1—H1D	117.6
C7—N1—H1A	110.7	O1—C6—O2	124.1 (2)
C7—N1—H1B	110.0	O1—C6—C2	119.0 (2)
H1A—N1—H1B	114.6	O2—C6—C2	116.87 (19)
C7—N1—H1C	109.6	N1—C7—C7^i^	110.1 (2)
H1A—N1—H1C	107.1	N1—C7—H7B	109.6
H1B—N1—H1C	104.5	C7^i^—C7—H7B	109.6
C5—N2—C1	116.2 (2)	N1—C7—H7A	109.6
C4—C3—C2	119.6 (2)	C7^i^—C7—H7A	109.6
C4—C3—H3A	120.2	H7B—C7—H7A	108.2
C2—C3—H3A	120.2	C5—C4—C3	118.5 (2)
N2—C5—C4	124.1 (2)	C5—C4—H4A	120.7
N2—C5—H5A	118.0	C3—C4—H4A	120.7
C4—C5—H5A	118.0		
			
C1—C2—C3—C4	0.2 (3)	C3—C2—C6—O1	1.0 (3)
C6—C2—C3—C4	−179.1 (2)	C1—C2—C6—O1	−178.3 (2)
C1—N2—C5—C4	−0.7 (4)	C3—C2—C6—O2	−178.8 (2)
C5—N2—C1—C2	1.0 (4)	C1—C2—C6—O2	1.9 (3)
C3—C2—C1—N2	−0.7 (4)	N2—C5—C4—C3	0.3 (4)
C6—C2—C1—N2	178.6 (2)	C2—C3—C4—C5	0.0 (4)

Symmetry codes: (i) −*x*+1, −*y*+1, −*z*+1.

### Hydrogen-bond geometry (Å, °)

**Table:**

*D*—H···*A*	*D*—H	H···*A*	*D*···*A*	*D*—H···*A*
N1—H1B···N2^ii^	0.90	2.24	2.971 (3)	138
N1—H1C···O1^iii^	0.90	1.85	2.729 (3)	164
N1—H1A···O2	0.90	1.83	2.711 (3)	166

Symmetry codes: (ii) −*x*+1, *y*−1/2, −*z*+3/2; (iii) *x*+1, *y*, *z*.

## References

[bb1] Aminabhavi, T. M., Biradar, N. S. & Patil, S. B. (1986). *Inorg. Chim. Acta*, **125**, 125–128.

[bb2] Fu, D.-W., Dai, J., Ge, J.-Z., Ye, H.-Y. & Qu, Z.-R. (2010). *Inorg. Chem. Commun.* **13**, 282-285.

[bb3] Rigaku (2005). *CrystalClear* Rigaku Corporation, Tokyo, Japan.

[bb4] Sheldrick, G. M. (2008). *Acta Cryst.* A**64**, 112–122. 10.1107/S0108767307043930 18156677

